# Complex Structure of OspI and Ubc13: The Molecular Basis of Ubc13 Deamidation and Convergence of Bacterial and Host E2 Recognition

**DOI:** 10.1371/journal.ppat.1003322

**Published:** 2013-04-25

**Authors:** Panhan Fu, Xiaoqing Zhang, Mengmeng Jin, Li Xu, Chong Wang, Zongping Xia, Yongqun Zhu

**Affiliations:** Life Sciences Institute, Zhejiang University, Hangzhou, Zhejiang, China; Osaka University, Japan

## Abstract

Ubc13 is an important ubiquitin-conjugating (E2) enzyme in the NF-κB signaling pathway. The *Shigella* effector OspI targets Ubc13 and deamidates Gln100 of Ubc13 to a glutamic acid residue, leading to the inhibition of host inflammatory responses. Here we report the crystal structure of the OspI-Ubc13 complex at 2.3 Å resolution. The structure reveals that OspI uses two differently charged regions to extensively interact with the α1 helix, L1 loop and L2 loop of Ubc13. The Gln100 residue is bound within the hydrophilic catalytic pocket of OspI. A comparison between Ubc13-bound and wild-type free OspI structures revealed that Ubc13 binding induces notable structural reassembly of the catalytic pocket, suggesting that substrate binding might be involved in the catalysis of OspI. The OspI-binding sites in Ubc13 largely overlap with the binding residues for host ubiquitin E3 ligases and a deubiquitinating enzyme, which suggests that the bacterial effector and host proteins exploit the same surface on Ubc13 for specific recognition. Biochemical results indicate that both of the differently charged regions in OspI are important for the interaction with Ubc13, and the specificity determinants in Ubc13 for OspI recognition reside in the distinct residues in the α1 helix and L2 region. Our study reveals the molecular basis of Ubc13 deamidation by OspI, as well as a convergence of E2 recognition by bacterial and host proteins.

## Author Summary

The Gram-negative pathogenic bacterium *Shigella* infects human intestinal epithelium cells and causes severe inflammatory colitis (bacillary dysentery). *Shigella* harbors an approximately 220-kb virulence plasmid that encodes a type III secretion system (T3SS) protein secretion apparatus and many effector proteins. Using the T3SS, *Shigella* delivers the effector proteins into the host cells, targeting key signal molecules and manipulating the host physiological processes and thereby promoting infection and multiplication. OspI, a newly identified *Shigella* effector, targets the host Ubc13 protein, a critical ubiquitin-conjugating enzyme in the NF-κB signaling pathway. OspI deamidates Gln100 of Ubc13 to a glutamic acid residue, thereby disrupting TRAF6-catalyzed polyubiquitination and dampening host inflammatory responses. However, the structural mechanism of this specific deamidation is unclear. Through crystallography, we have determined the structure of the OspI-Ubc13 complex. The structure illustrates how OspI interacts with Ubc13 and how Ubc13 induces conformational changes in OspI. Combining structural analysis and biochemical assays, we revealed how OspI distinguishes Ubc13 from other ubiquitin conjugating enzymes and found that OspI binds to the same surface region on Ubc13 as host TRAF6, CHIP and OTUB1. Our study sheds light on the molecular mechanism of Ubc13 deamidation by OspI and provides new insights into E2 recognition by bacterial and host proteins.

## Introduction

Ubiquitination is a post-translational protein modification involved in many important cellular processes, such as signal transduction, protein degradation and vesicle trafficking [1]. Ubiquitination is catalyzed by a combined cascade of three enzymes: a ubiquitin-activating enzyme (E1), a ubiquitin-conjugating enzyme (E2) and a ubiquitin ligase (E3). Ubiquitin is activated by E1 before being transferred onto the catalytic cysteine residue of E2 through a thioester bond. E3 bridges the specific substrate and ubiquitin-charged E2, catalyzing ubiquitin-chain formation on the substrate. As they couple the upstream activation of ubiquitin and the downstream modification events, E2 enzymes play a central role in the enzymatic cascade of ubiquitination [2]. However, compared to E3 ligases, whose regulation has been extensively studied, little is known about how E2 enzymes are regulated in cells. Among the approximately 39 E2 enzymes encoded in the human genome [3], Ubc13, also known as UBE2N, is a unique E2 enzyme because it forms a heterodimeric complex with Mms2/Uev1a, which binds to the central β-sheet region of Ubc13 [4] [5]. Although involved in many other cellular processes, Ubc13 has been primarily studied for its critical role in regulating the NF-κB signaling pathway [6]. Upon stimulation from receptors, Ubc13 and the RING-type E3 ligase TRAF6 catalyze the synthesis of long Lys63-linked polyubiquitin chains to activate two important downstream kinase complexes, the TAK1 complex and IKK complex, to mediate inflammatory responses [7].

*Shigella*, a Gram-negative pathogenic bacterium, causes human shigellosis by invading the intestinal epithelium cells after ingestion. *Shigella* delivers a subset of effectors into host cells via a specifically evolved type III secretion system, modulating the cellular processes and promoting infection and multiplication [8]. The key signaling molecules essential for host defenses are frequent targets of these effectors. The *Shigella* OspF effector exhibits phosphothreonine lyase activity and irreversibly dephosphorylates MAPKs, inhibiting the MAPK signaling pathway [9], [10]. IpaH9.8 and IpaH4.5, which belong to a new IpaH family of ubiquitin E3 ligases [11], [12], [13], [14], inhibit the NF-κB signaling pathway by ubiquitinating NEMO and p65, respectively [15], [16]. The *Shigella* VirA effector inactivates Rab1 with TBC-like GAP activity, inhibiting the host autophagy-mediated defense [17].

A recent study revealed that a newly identified *Shigella* effector, OspI, targets the host Ubc13 and deamidates Gln100 to a glutamic acid residue, leading to the disruption of TRAF6-catalyzed polyubiquitination [18]. The disruption of TRAF6 polyubiquitination suppresses the diacylglycerol-CBM (CARD-BCL10-MALT1 complex)-TRAF6-NF-κB signaling pathway and dampens the host inflammatory responses [18]. However, the structural mechanisms underlying the deamidation and how a bacterial effector protein selectively recognizes a ubiquitin conjugating enzyme are unclear. Here, we describe the crystal structure of the OspI-Ubc13 complex at 2.3 Å resolution. OspI targets Ubc13 via extensive interactions, and Ubc13 binding remodels the structure of OspI for catalysis. Although the structures of OspI alone and other papain-like proteins have been reported, the direct observation of the interactions between a papain-like protein and an E2 enzyme is, to our knowledge, unprecedented. Structural analysis of the Ubc13 complexes with OspI, TRAF6, CHIP and OTUB1 revealed that OspI binds to the same surface region on Ubc13 as the host proteins. Our biochemical studies further analyzed the specificity determinants in Ubc13 for OspI recognition.

## Results

### Overall Structure of the OspI-Ubc13 Complex

To generate the OspI-Ubc13 complex, we mutated the catalytic residue Cys62 of OspI [18] to alanine (C62A) and purified the mutant protein to homogeneity. Herein, OspI refers to the C62A mutant unless the wild type is explicitly denoted. Purified OspI was mixed with equimolar amounts of Ubc13 and incubated overnight at 4°C. After incubation, we subjected the mixture to size exclusion chromatography and found a peak containing a 1∶1 heterodimeric complex of OspI and Ubc13. We used the equimolar mixture to set up crystallization trials. The obtained crystals of the OspI-Ubc13 complex were confirmed using SDS-PAGE (Figure S1). The structure was solved using the molecular replacement method and finally refined to 2.3 Å with Rwork/Rfree values of 21.0%/24.8% and high-quality geometry. The details of data collection and refinement statistics are listed in Table 1.

**Table 1:** Data Collection and Refinement Statistics.

Data collection	
Space group	P321
Cell dimensions	
a, b, c (Å)	119.06, 119.06, 69.74
α, β, γ (°)	90.00, 90.00, 120.00
Wavelength	0.9785
Resolution (Å)^a^	50-2.30 (2.34-2.30)
*R*_sym_ or *R*_merge_	0.078 (0.514)
*I*/σ*I*	56.65 (7.4)
Completeness (%)	99.8 (100)
Redundancy	12.2 (12.6)

Refinement	
Resolution (Å)	50-2.30
No. reflections	24,433
*R*_work/_ *R*_free_(%)^b^	21.0/24.8
No. atoms	
Protein	2,689
Water	155
B-factors(Å^2^)	
Protein	54.47
Water	48.52
R.m.s deviations	
Bond lengths (Å)	0.006
Bond angles (°)	1.232
Ramachandran Plot	
Preferred regions(%)	95.9
Allowed regions(%)	4.1

a The data for the highest resolution shell are shown in parentheses.

b R_free_ is calculated using 10% of the total number of reflections.

**Figure 1:**
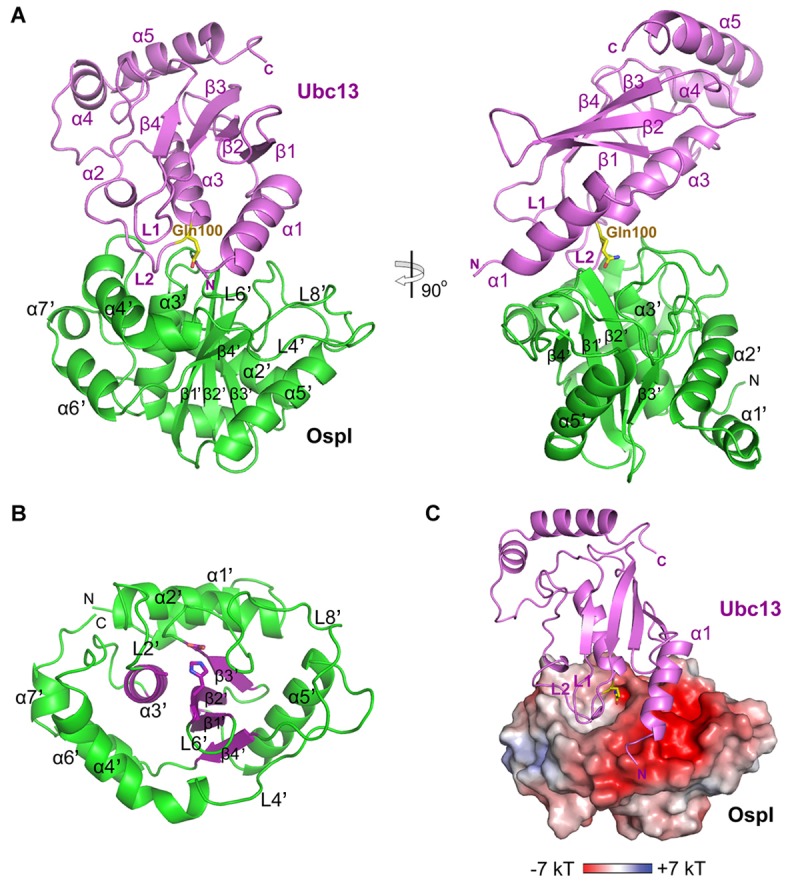
Crystal Structure of the OspI-Ubc13 Complex. (A) Overall structure of the OspI-Ubc13 complex. OspI and Ubc13 are colored in green and purple, respectively. The glutamine residue Gln100 of Ubc13 is shown as yellow sticks. (B) Structure of OspI in the complex. The core secondary structure elements of the papain-like fold (α3′ and β1′–β4′) in OspI are highlighted in dark purple. The residues Ala62, His145 and Asp160 in the catalytic triad are shown as sticks. (C) The electrostatic surface potential of OspI with the bound Ubc13 shown as ribbons. The electrostatic potential is calculated with Adaptive Poisson-Boltzmann Solver (APBS) and illustrated (−7 to +7 kT) with PyMOL.

The overall structure of the OspI-Ubc13 complex assembles into an oblique “L” shape (Figure 1A), with Ubc13 bound on the top surface of OspI. In the complex, Ubc13 adopts the typical UBC fold of E2 enzymes [2], [19], with an elongated structure composed of four α-helices (α1, α3–α5), a central four-stranded β-sheet (β1–β4) and a 3_10_-helix (α2). We designated the β3–β4 linking loop as L1 and the loop between α2 and α3 as L2 (Figure 1A). Structural comparison revealed that the overall architecture of Ubc13 in the complex is very similar to previously determined Ubc13 structures [4], [20], [21] (Figure S2A). However, the N-terminal α1 helix undergoes notable conformational changes upon OspI binding. As they are largely involved in the interactions with OspI (details below), the extreme N-terminal four residues of the α1 helix are rotated downward to be in contact with OspI, and the C-terminus moves slightly toward the main body of Ubc13 (Figure S2A).

Similarly to the non-complexed wild-type alone structure [18], OspI in the complex with Ubc13 adopts a cylindrical single-domain architecture with seven α-helices (α1′–α7′) and four β-strands (β1′–β4′) (Figure 1A and 1B). Interestingly, in addition to the previously described AvrPphB [18], our structural homolog search with the Dali server [22] revealed that OspI has definite structural similarities to the catalytic domains of several host papain-like deubiquitinating enzymes, including CYLD, USP7, USP14, USP8 and USP21 (Figure S3). These deubiquitinating enzymes have conserved Cys-His-Asp catalytic triads to hydrolyze the mono- or poly-ubiquitin chains from substrates [23], [24], [25], [26], [27]. The structural homolog search also revealed that OspI has low structural similarities to the recently identified ubiquitin/NEDD8 deamidases CHBP from *Burkholderia pseudomallei* and Cif from enteropathogenic *Escherichia coli* (EPEC) [28], [29], [30] (Figure S3). CHBP and Cif adopt a papain-like fold similar to OspI and catalyze the deamidation on the conserved Gln40 residue in ubiquitin and NEDD8 with the Cys-His-Gln catalytic triads. However, only the core structures of the papain-fold in CHBP and Cif can be superimposed with that of OspI. The overall architecture of OspI is largely different from the extended structures of CHBP and Cif (Figure S3).

### Interaction Interface between OspI and Ubc13

In the complex, OspI and Ubc13 contact each other via a large interface with a burial surface area of 976.1 Å^2^, which covers 11% of the total accessible surface of OspI (Figure 1C). The OspI-binding region in Ubc13 includes the α1 helix, L1 and L2 loops, far from the Mms2/Uev1-binding β-sheet region [4], which suggests that OspI binding does not affect the interaction between Ubc13 and Mms2/Uev1a. The surface electrostatic potential calculation revealed that the Ubc13-binding surface on OspI covers two differently charged regions: an acidic, negatively charged region and an open, hydrophobic pocket (Figure 1C). The negatively charged region is comprised of residues from the L4′, L6′ and L8′ loops (Figure 1B). This acidic patch complementarily binds to the positively charged α1 helix of Ubc13. The hydrophobic pocket of OspI comprises residues from α3′, α4′, L6′ and β2′ and binds to the L1 and L2 loops of Ubc13 (Figure 1B and 1C).

**Figure 2:**
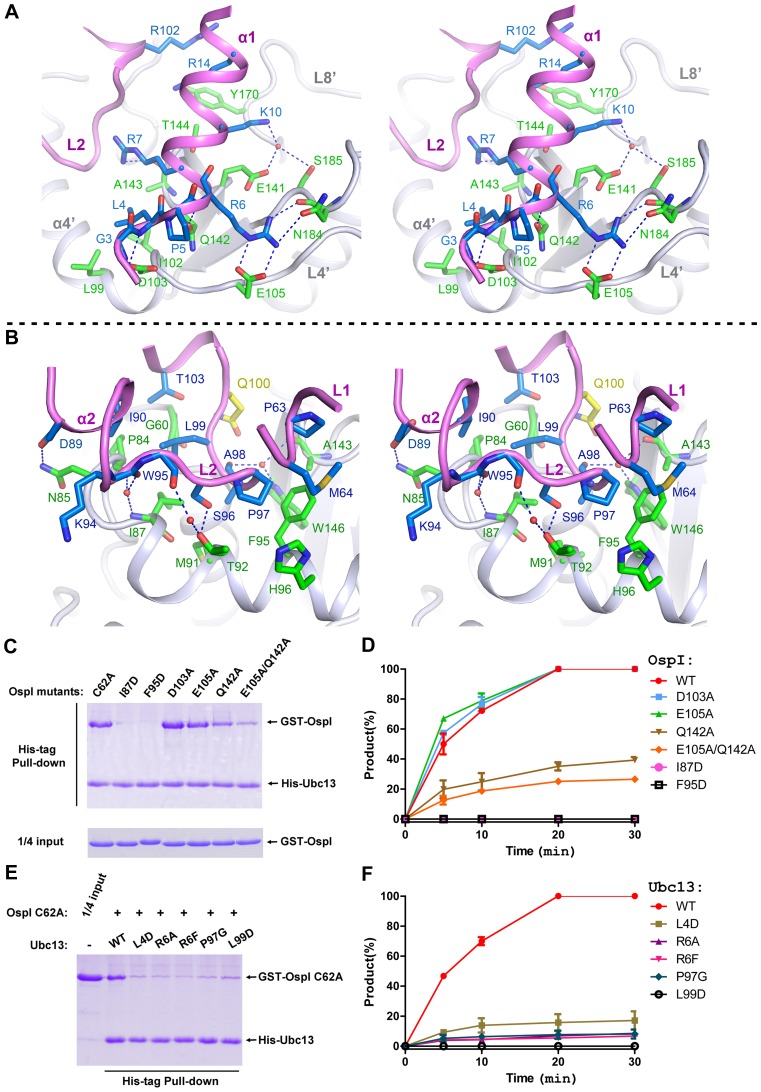
Interactions between OspI and Ubc13. (A) Cross-eyed stereo view of the interactions between OspI with the α1 helix of Ubc13. OspI and Ubc13 are shown as ribbons in gray and purple, respectively. The residues of OspI and Ubc13 participating in the interactions are represented as sticks and colored in green and blue, respectively. The water molecules are represented as red spheres. The blue dashed lines indicate the hydrogen bonds. (B) Cross-eyed stereo view of the interactions between OspI with the L1 and L2 loops of Ubc13 colored as in (A). Only the main chain of Trp95 in the L2 loop of Ubc13 is shown for a clear view. (C) Effects of OspI mutations on Ubc13 interactions. The interactions of wild-type Ubc13 with GST-C62A variants were examined in His-tag pull-down assays. (D) Quantitative analysis of the deamidation activities of mutant OspI proteins. The percent product (Ubc13-E100) formed as a function of time by wild-type OspI and its mutant proteins is plotted. (E) Effects of Ubc13 mutations on OspI interactions. The interactions of GST-C62A with Ubc13 variants were examined in His-tag pull-down assays. (F) Quantitative analysis of the deamidation activities of wild-type OspI on mutant Ubc13 proteins. The percent product (Ubc13-E100) of variant mutant Ubc13 proteins formed as a function of time by wild-type OspI is plotted. All assays were repeated more than 3 times. The error bars represent the standard deviations.

The interactions between the α1 helix of Ubc13 and the negatively charged region of OspI involve an extensive network of hydrogen bonds (Figure 2A). Arg6^Ubc13^ is bound to a cleft of OspI and contacted by Glu105^OspI^, Gln142^OspI^ and Asn184^OspI^ through five hydrogen bonds. Lys10^Ubc13^ is dragged into the acidic patch by Glu141^OspI^ and Ser185^OspI^. The residues Gly3^Ubc13^, Leu4^Ubc13^ and Arg7^Ubc13^ are contacted by Asp103^OspI^ and Ala143^OspI^. In addition to hydrogen bonds, the interactions between OspI and the α1 helix also include Van der Waals contacts and hydrophobic interactions. Thr144^OspI^ and Tyr170^OspI^ bind to the α1 helix from the bottom. Gln142^OspI^ interacts with Ubc13 by wedging into the cleft between Leu4^Ubc13^ and Arg6^Ubc13^. Leu99^OspI^ and Ile102^OspI^ contact the side chain of Leu4^Ubc13^. Compared to the early Ubc13 structures [4], [20], [21], the extreme N-terminus of the α1 helix is re-oriented upon OspI binding (Figure S2). Gln142^OspI^ wedging seems to play a critical role in the conformational change because Gln142^OspI^ wedging induces the redirection of Pro5^Ubc13^, which forces Leu4^Ubc13^ to move downward (Figure S2B). The subsequent interactions of Asp103^OspI^ with Gly3^Ubc13^ and Leu4^Ubc13^ likely stabilize the new orientation.

The binding of the L1 and L2 loops of Ubc13 in the open pocket of OspI mainly involves hydrophobic interactions (Figure 2B). Ile87^OspI^ and Phe95^OspI^ clamp the L2 loop of Ubc13 from two sides at the bottom through interactions with Pro97^Ubc13^ and Ala98^Ubc13^. Leu99^Ubc13^ is locked by Pro84^OspI^ and Ile87^OspI^. Pro63^Ubc13^ and Met64^Ubc13^ in the L1 loop are bound by Phe95^OspI^ and His96^OspI^. Met91^OspI^ and Trp146^OspI^ participate in the hydrophobic interactions at the bottom of the pocket. Additionally, one direct and nine water-mediated hydrogen bonds peripherally stabilize the hydrophobic interactions. Four residues vicinal to the L2 loop of Ubc13, including Asp89^Ubc13^, Ile90^Ubc13^, Arg102^Ubc13^ and Thr103^Ubc13^, are also involved in the interaction with OspI (Figure 2A and 2B).

### Mutagenesis Analysis

To assess the roles of the observed interactions, we generated a panel of missense mutations of OspI. We examined the interactions between OspI and Ubc13 by using His-tag mediated pull-down assays and performed glutamine deamidation assays to evaluate the deamidase activities of the OspI variants (details in Materials and Methods). All of the mutant proteins behave well in terms of protein solubility and stability. The single mutations D103A and E105A had little effect on the Ubc13-binding and deamidation activities of OspI (Figure 2C and 2D), likely due to the extensive charge interactions between OspI and the α1 helix. A Q142A mutant could not efficiently bind to Ubc13 and exhibited a relatively low deamidation activity, suggesting that the Gln142 protrusion into the cleft between Leu4 and Arg6 of Ubc13 is important for OspI binding to the α1 helix. Similar to Q142A, the double mutation E105A/Q142A not only greatly disrupted Ubc13-binding ability but also reduced the deamidase activity of OspI. Single mutations (I87D and F95D) of the OspI residues for binding L1 and L2 loops completely eliminated Ubc13-binding ability as well as deamidase activity (Figure 2C–D), indicating that the hydrophobic interactions between OspI and the L1 and L2 loops are critical for binding Ubc13. The dysfunction of the OspI mutants, including Q142A, E105A/Q142A, I87D and F95D, suggests that both the negatively charged region and the hydrophobic pocket in OspI for the binding the α1 helix and L1 and L2 loops are required for the recognition and deamidation of Ubc13. Consistently with this idea, mutations of the OspI-binding residues in the α1 helix and L2 loop of Ubc13, including L4D, R6A, R6F, P97G and L99D, completely abolished the interaction between Ubc13 and OspI (Figure 2E). Wild-type OspI was also unable to efficiently catalyze the deamidation on the mutant Ubc13 proteins (Figure 2F).

**Figure 3:**
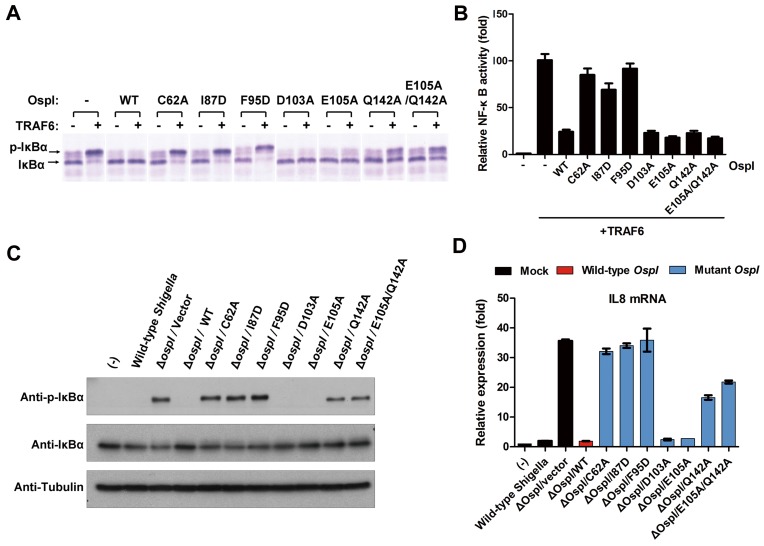
Effects of OspI Variants on NF-κB Activation. (A) Inhibition of TRAF6-induced phosphorylation of IκBα by OspI variants in cell-free extracts. The U937 S100 cell extracts were incubated with recombinant TRAF6, the indicated OspI mutant proteins and ATP at 30°C for 1 h. The samples were analyzed by immunoblotting and using the IκBα antibody. (B) NF-κB luciferase reporter assays in HEK293 cells transfected with TRAF6 and the indicated OspI variants. (C) The phosphorylation of IκBα in HeLa cells infected with Δ*ospI Shigella* complemented with the indicated *OspI* mutant plasmids. (D) IL8 expression level indicating NF-κB activation in HeLa Cells infected with Δ*ospI Shigella* harboring the indicated *OspI* mutant plasmids.

We further used the U937 S100 cell extract as an *in vitro* reconstitution system to test the effects of the OspI mutations on the NF-κB signaling pathway (Figure 3A). Consistent with our above-described results, the D103A and E105A mutants inhibited the TRAF6-induced phosphorylation of IκBα *in vitro* as wild-type OspI. The I87D, F95D, Q142A and E105A/Q142A mutations largely abolished the inhibitory effect. The *in vivo* effects of the OspI mutants were examined by using NF-κB luciferase reporter assays in HEK293 cells (Figure 3B). Similar to the C62A mutant, I87D and F95D mutants demonstrated a severely reduced ability to inhibit the TRAF6-induced NF-κB activation. D103A and E105A presented similar inhibitory capabilities to wild-type OspI. Because Q142A and E105A/Q142A still inhibited TRAF6-induced NF-κB activation in the luciferase reporter assays (Figure 3B), likely due to their residual deamidation activities and long-lasting expression in cells, we further constructed an *OspI*-deletion *Shigella* strain and performed infection assays to validate the physiological effects of the OspI variants. Complementing the Δ*ospI* mutant with the Q142A or E105A/Q142A gene did not efficiently suppress IκBα-phosphorylation and *IL8* mRNA production induced by Δ*ospI* infection as with the wild-type *OspI* gene (Figure 3C, 3D and S4), which is consistent with the *in vitro* studies. The I87D and F95D genes also could not rescue the infection function of the Δ*ospI* mutant. Therefore, both the negatively charged region and the hydrophobic pocket in OspI for binding of the α1 helix and L1 and L2 loops are indeed necessary for the recognition and deamidation of Ubc13.

### The Catalytic Pocket and Its Structural Reassembly

**Figure 4:**
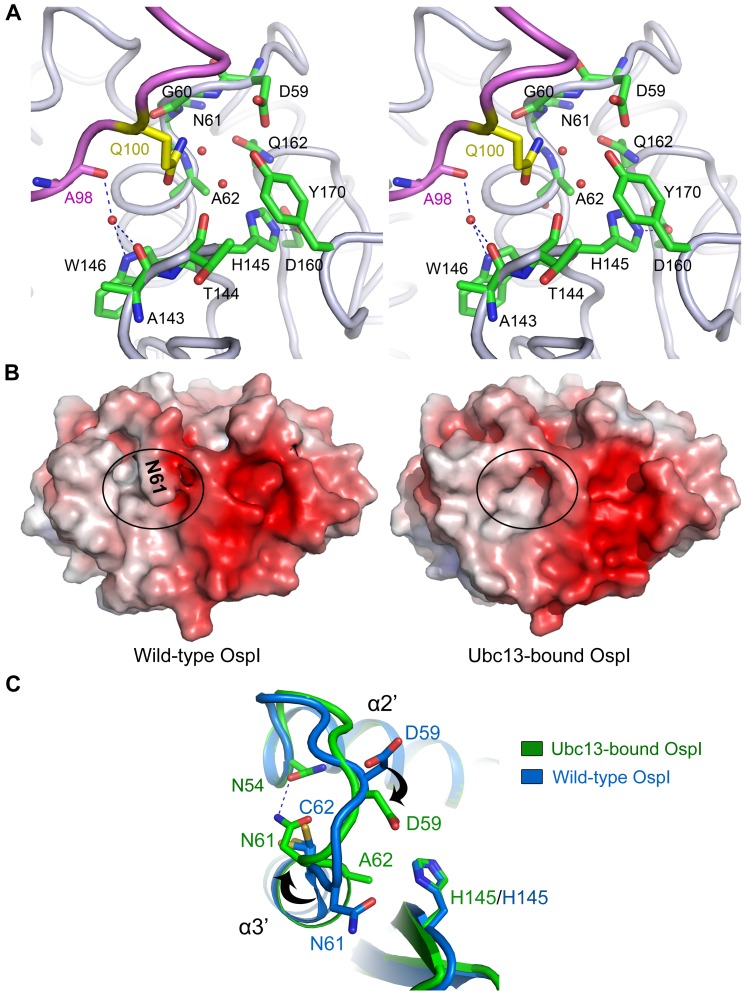
The Catalytic Pocket of OspI and its Structural Reassembly. (A) Cross-eyed stereo view of the Gln100 binding in the catalytic pocket of OspI. Ubc13 (purple) and OspI (gray) are shown as loops. Gln100 and the residues forming the catalytic pocket of OspI are shown as sticks and colored in yellow and green, respectively. The red spheres represent water molecules. The blue dashed lines indicate the hydrogen bonds. (B) Comparison of the catalytic pockets in wild-type (left) and Ubc13-bound OspI (right). Both structures are represented as surfaces. The catalytic pockets in the two structures are highlighted with black circles. The Asn61 shielding the catalytic pocket in wild-type OspI is labeled in black. (C) Conformational changes of the catalytic pocket of OspI upon Ubc13 binding. Structures of Ubc13-bound and wild-type OspI are shown as ribbons and colored in green and blue, respectively. The hydrogen bond between Asn61 and Asn54 in Ubc13-bound OspI is shown as a blue dashed line. The conformational changes of Asn61 and Asp59 are highlighted with black arrows.

The binding of the α1 helix, L1 and L2 loops of Ubc13 extends Gln100 into the catalytic pocket of OspI. The catalytic pocket is largely hydrophilic and is comprised of ten residues (Figure 4A). Asp59, Gly60, Ala143, Thr144 and Tyr170 form the edge of the pocket, and the bottom is formed by Ala62, His145, Trp146, Asp160 and Gln162. In addition to Gln100 of Ubc13, there are two water molecules in the catalytic pocket, suggesting that water molecules have the opportunity to enter the catalytic pocket of OspI to participate in the deamidation reaction. The structural comparison of the catalytic pockets of OspI and AvrPphB revealed that the Gln162 residue in OspI occupies an analogous position to the oxyanion hole residue Asn93 in AvrPphB [31] (Figure S5B and S5C), which suggests that Gln162 is also involved in the catalysis of OspI by forming the oxyanion hole. The distance between Gln100 of Ubc13 and Ala62 of OspI is only approximately 4.5 Å, indicating that Gln100 of Ubc13 can be bound appropriately into the catalytic pocket in wild-type OspI during the deamidation. The structural similarities between OspI and AvrPphB suggest that the catalytic mechanism of OspI is similar to that of the papain-like cysteine protease [31], [32]. Asp160 orients His145 to form a thiolate-imidazolium ion pair with Cys62 to activate the catalytic residue. The activated Cys62 attacks the carbon atom in the δ-carboxamide of Gln100 of Ubc13 with the thiolate to release the NH_3_ product. Then, a water molecule nucleophilically attacks the covalent acyl-Cys62 intermediate to produce a glutamic acid residue.

Compared to the wild-type alone structure [18], OspI in the complex undergoes notable conformational changes (Figure S5). Upon Ubc13 binding, Phe95 and His96 of OspI move up to form a hydrophobic pocket for interaction with the L1 and L2 loops (Figure S5A). The movement of Phe95 and His96 refolds two 3_10_-helices (3_10_^−1a^ and 3_10_^−1b^) and a short helix (α4) into a long, integrated helix (α4′ in the Ubc13-bound OspI) (Figure S5A). A comparison between the Ubc13-bound and wild-type OspI structures revealed that the catalytic pocket in wild-type OspI is completely shielded by the Asn61 residue (Figure 4B). The active residue Cys62 of wild-type OspI does not form a thiolate-imidazolium ion pair with His145 [18] (Figure 4C) and could not be aligned with the active site Cys98 of AvrPphB in the structural superimposition (Figure S5B). Upon Ubc13 binding, Asn61 rotates backward by approximately 180° to be bound by Asn54 with a hydrogen bond, which induces the repositioning of Ala62 and opens the catalytic pocket (Figure 4C and S5). Additionally, Asp59 of OspI rotates by approximately 110° to form the edge of the catalytic pocket. After the structural reassembly, the repositioned Ala62 can be well superimposed with Cys98 of AvrPphB [31] (Figure S5C), which suggests that Ala62 (Cys62 in wild-type OspI) is placed at the proper catalytic position upon Ubc13 binding. The reassembly of the catalytic pocket suggests that Ubc13 binding-induced conformational changes are involved in the catalysis of OspI.

### Specificity Determinants in Ubc13 for OspI Recognition

**Figure 5:**
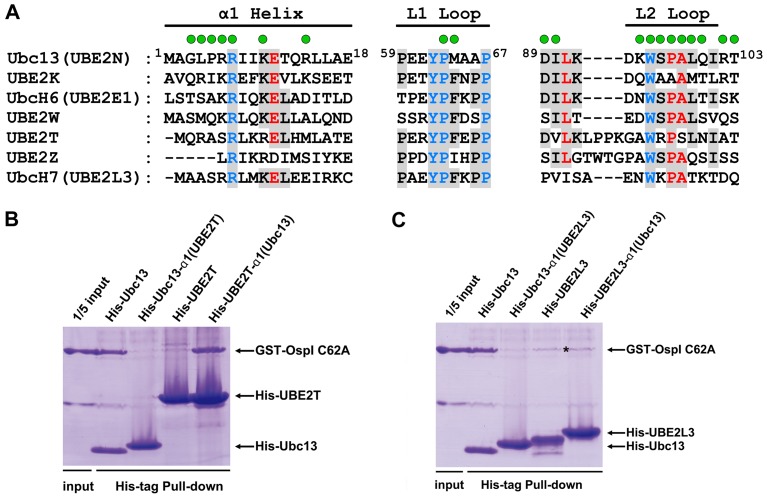
Specificity Determinants for Ubc13 in OspI Recognition. (A) Multiple sequence alignment of the α1 helices, L1 and L2 loops from different E2 enzymes. The conserved residues are colored in blue, red and black with a gray background. The OspI binding residues in Ubc13 are highlighted above the Ubc13 sequence with green circles. (B) The interactions of OspI with wild-type Ubc13, Ubc13-α1(UBE2T) (Ubc13 mutant with exposed residues in the α1 helix replaced by those of UBE2T), wild-type UBE2T and UBE2T-α1(Ubc13) (UBE2T mutant with exposed residues in the α1 helix replaced by those of Ubc13). (C) The interactions of OspI with wild-type UBE2L3 and UBE2L3-α1(Ubc13) (UBE2L3 mutant with exposed residues in the α1 helix replaced by those of Ubc13). The nonspecific binding of GST-C62A to Ni-NTA beads in the pull-down assays is marked by an asterisk.

Although all E2 enzymes adopt similar UBC-fold structures [2], [19], OspI is specific for Ubc13. In the complex structure, OspI interacts with Ubc13 via binding the α1 helix, L1 loop and L2 region (including the L2 loop and its adjacent four residues), indicating that these regions determine the specificity for Ubc13. Sequence alignment revealed that the sequences of Ubc13 and other E2s are highly conserved in the L1 loop and moderately conserved in the L2 region but differ significantly in the α1 helix (Figure 5A). Therefore, it is likely that the α1 helix of Ubc13 is critical for OspI discrimination between the host E2 enzymes. Consistent with this prediction, Ubc13 proteins with mutations in the α1 helix, such as L4D, R6A and R6F, cannot be efficiently recognized by OspI, as demonstrated in the *in vitro* pull-down assays (Figure 2E). UBE2T is an E2 enzyme belonging to the same subfamily [3] and has conserved sequences in the L2 region with Ubc13. To further test our prediction, we replaced the surface residues in the α1 helix of UBE2T with those of Ubc13 and reciprocally replaced the exposed residues in the α1 helix of Ubc13 with those of UBE2T. Consistent with our prediction, replacing the α1 helix of UBE2T with that of Ubc13 allowed OspI binding, although OspI could not catalyze the deamidation reaction as there is no counterpart glutamine residue in UBE2T (Figure 5B). Ubc13 with the α1 helix from UBE2T could no longer be recognized by OspI. These results indicate that recognition of the α1 helix by OspI is critical for the specificity for Ubc13. The OspI-binding residues Gly3, Leu4, Pro5, Arg6 and Arg14, in the α1 helix of Ubc13, are distinct from other E2 enzymes but are required for OspI recognition and deamidation (Figure 2E, 2F and S6), suggesting that the interactions with these residues is the molecular basis of the specific recognition of the α1 helix.

The L2 region of Ubc13 required for the interaction with OspI (Figure 2E) is conserved in many E2s, but this region differs from those in a few E2 enzymes, such as UBE2L3 (also named UbcH7) (Figure 5A). To better understand how OspI discriminates between Ubc13 and UBE2L3, and whether the L2 region has a role in the specificity for Ubc13, we further swapped the surface residues in the α1 helices of Ubc13 and UBE2L3. As shown in our pull-down assays (Figure 5C), even though the α1 helix of UBE2L3 was replaced with that of Ubc13, UBE2L3 still could not be recognized by OspI, suggesting that recognition of the α1 helix by OspI is not sufficient for the specificity of Ubc13, and the L2 region required for OspI recognition (Figure 2E) has an important role in the discrimination of Ubc13 from other E2 enzymes. Comparison of the sequences of UBE2L3, Ubc13 and UBE2T revealed that the residues Leu99, Arg102 and Thr103 in the L2 region of Ubc13 are similar to the L2 region residues in UBE2T but distinct from the residues in UBE2L3 (Figure 5A). These residues are all required for OspI recognition and deamidation (Figure 2E, 2F and S6), suggesting that these residues determine the specific recognition of the L2 region by OspI. Taken together, these results indicate that the specificity determinants for Ubc13 in OspI recognition reside in the distinct residues of the α1 helix and L2 region.

### The Same Binding Surface in Ubc13 for OspI and Host Proteins

**Figure 6:**
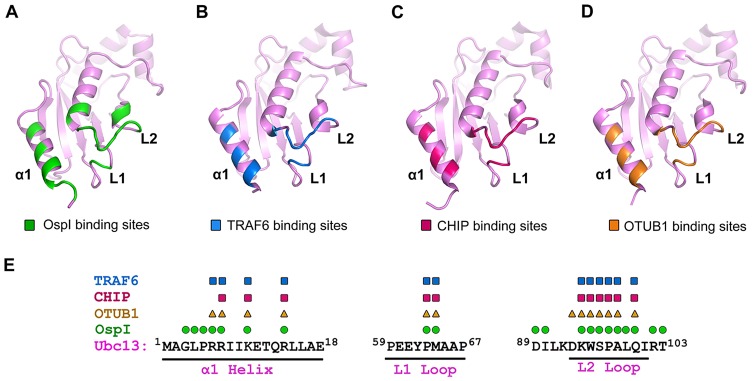
The Binding Regions in Ubc13 for OspI and Host Proteins. (A–D) Structures of Ubc13 in complexes with OspI, TRAF6 (pdb ID: 3HCT), CHIP (pdb ID: 2C2V) and OTUB1 (pdb ID: 4DHI) in the same orientation and shown as purple-colored cartoons. The binding regions for OspI, TRAF6, CHIP and OTUB1 in Ubc13 are highlighted in green (A), blue (B), red (C) and orange (D), respectively. (E) Sequence analysis of the Ubc13 residues bound by TRAF6, CHIP, OTUB1 and OspI, which are marked above the Ubc13 sequence with blue rectangles, red rectangles, orange triangles and green circles, respectively.

The specific recognition of Ubc13 in host cells has been well characterized by the structural studies of Ubc13 complexes with ubiquitin E3 ligases, TRAF6 and CHIP [20], [21]. TRAF6 is a RING-type E3 ligase and interacts with Ubc13 by using its RING domain [20]. The TRAF6-binding region in Ubc13 covers the α1 helix, L1 and L2 loops (Figure 6 and S7). The binding of TRAF6 to Ubc13 highly stimulates the synthesis of long Lys63-linked polyubiquitin chains to activate TAK1 and IKK complexes [7]. CHIP is a U-box E3 ligase and regulates the growth hormone receptor endocytosis by catalyzing Lys63-linked polyubiquitin chain formation [33]. Similar to TRAF6, CHIP binds to the α1 helix, L1 and L2 loops of Ubc13 (Figure 6C) via its U-box, which is structurally related to the RING domain [21] (Figure S7C). Additionally, recent studies have revealed a specific interaction between Ubc13 and the deubiquitinating enzyme OTUB1 [34], [35]. OTUB1 binding to Ubc13 interferes with the interactions of donor ubiquitin and TRAF6 with the E2 enzyme, leading to the inhibition of Lys63-linked polyubiquitin chain synthesis in DNA damage response [34]. Like TARF6 and CHIP, OTUB1 binds to Ubc13 through the α1 helix, L1 and L2 loops (Figure 6A–D). As a pathogenic bacterial effector, OspI specifically targets Ubc13 and deamidates the Gln100 residue to inhibit the host inflammatory responses. Interestingly, although the overall structure and function of OspI are completely different from those of host TRAF6, CHIP and OTUB1, OspI also recognizes Ubc13 by interacting with the α1 helix, L1 and L2 loops (Figure 6A). Most of the OspI-binding residues in Ubc13 are also the binding sites for the host E3 ligases and the deubiquitinating enzyme (Figure 6E), which suggests that OspI targets the same surface on Ubc13 as host proteins do. The existence of overlapping binding sites in Ubc13 suggests that a convergence exists in the E2 recognition by bacterial and host proteins. The additional interactions between OspI and the other residues of Ubc13 would enhance the OspI recognition efficiency and specificity for Ubc13.

### Occluding the TRAF6 Binding on Ubc13 by OspI

The same binding region of OspI and TRAF6 in Ubc13 raises the possibility that OspI can directly occlude TRAF6 binding on Ubc13 to inhibit the TRAF6-catalyzed polyubiquitination, as has been previously described for OTUB1 [34], [35]. To test the hypothesis, we performed *in vitro* ubiquitination assays by adding the C62A mutant protein into the reactions. When its concentration is near or higher than that of Ubc13, C62A efficiently inhibits the TRAF6-catalyzed polyubiquitination (Figure S8A). Adding an excess C62A into U937 S100 cell-free extract completely disrupts the TRAF6-induced phosphorylation of IκBα (Figure S8B). C62A could also slightly inhibit NF-κB activation in a dose-dependent manner in the luciferase reporter assays [18]. Thus, OspI indeed has the ability to occlude the TRAF6 binding on Ubc13 because of their identical binding surface. The inhibitory activity of C62A was much lower than that of the wild-type protein when they were at low concentration in the assays [18] (Figure 3), which suggests that the deamidase activity plays a central role in the function of OspI while occluding the TRAF6 binding on Ubc13 provides additional inhibition.

### Comparison with Other Glutamine Deamidases from Bacterial Pathogens

Five additional glutamine deamidases have been identified from various bacterial pathogens. CNF1, secreted by uropathogenic *Escherichia coli* (UPEC) into host cells, specifically targets RhoA GTPase to regulate the host cytoskeleton [36]. BLF1 (also known as BPSL1549), from *Burkholderia pseudomallei*, deamidates the host translation factor elF4A and abolishes its helicase activity, causing host translational inhibition [37]. VopC, from *Vibrio parahaemolyticus*, acts on Rac1 and Cdc42 for mediating pathogen invasion [38]. PMT, from *Pasteurella multocida*, causes the constitutive activation of G proteins by targeting G_αi2_ and G_αq_ [39]. CHBP, from *B. pseudomallei*, and Cif, from EPEC, deamidate ubiquitin/Nedd8 and induce host cell cycle arrest [28]. Among these glutamine deamidases, PMT, CHBP and Cif adopt the papain-like fold as OspI (Figure S9) and have similar Cys-His-Asp/Gln catalytic triads [28], [29], [30], [39]. However, BLF1, VopC and CNF1 have high structural or sequence similarities with each other and adopt the same CNF-C fold [36], [37], [38]. Unlike in OspI, the catalytic triads in BLF1, VopC and CNF1 only contain conserved cysteine and histidine residues. The third residues in their catalytic triads are variable (Figure S9A and S9B). Based on their structural folds, these bacterial pathogen glutamine deamidases should be classified into two types: CNF-C type and papain-like type.

Interestingly, although OspI, PMT, CHBP and Cif are largely different in overall structure (Figure S9), each of the proteins expresses the highest structural similarity to AvrPphB in their independent structural homolog search [29], [40], [41], which suggests that these papain-like deamidases from various bacterial pathogens might have evolved from a common ancestor. In addition, some common structural features can be found among these papain-like glutamine deamidases. OspI, PMT, CHBP and Cif all possess a similar hydrophilic pocket on their surfaces to accommodate the substrate glutamine residue (Figure S9). The catalytic residue Cys62 of OspI is buried in a deep glutamine-binding pocket and covalently bound by Cys65 with a disulfide bond [18]. The similar structure is also found in PMT [41]. The active cysteine residues of CHBP and Cif are only partially exposed at the edge of the glutamine-binding pocket (Figure S9D and S9E).

## Discussion

An emerging concept is that bacterial pathogens employ effector proteins to modulate the host ubiquitination pathway for promoting infection and multiplication [42]. It is important to characterize which bacterial effectors and how they manipulate host ubiquitin signaling. Our structure of the OspI-Ubc13 complex reveals the molecular basis of how a bacterial type III effector specifically recognizes and modulates a ubiquitin-conjugating enzyme. This is the first reported direct observation of interactions between a papain-like protein and an ubiquitin-conjugating E2 enzyme. Remarkably, the bacterial protein with a papain-like fold targets the ubiquitin-conjugating enzyme by binding to a region that is also used for interactions with host proteins, which suggests that a convergence exists in the recognition of the ubiquitin-conjugating enzyme by pathogenic bacterial and host proteins after their long-term co-evolution.

Given that a large number of papain-like proteins exist in eukaryotic cells, it is possible that some host papain-like proteins might function as glutamine deamidases to inactivate proteins in the ubiquitination pathway. A recent study revealed that the OUT-type deubiquitinating enzyme A20 regulates Ubc13 and UbcH5c to inhibit the E3 ligase activities of TRAF6, TRAF2 and cIAP1 [43]. Surprisingly, our structural homolog search using the Ubc13-bound OspI structure as the bait revealed that many host papain-like deubiquitinating enzymes have structural similarities with OspI. It will be very interesting to test whether some of these papain-like deubiquitinating enzymes can interact with or regulate E2 enzymes in host cells. A structural comparison of OspI, PMT, CHBP and Cif uncovered some common structural features of these bacterial papain-like glutamine deamidases. These common features should be helpful for us in structurally identifying new glutamine deamidases from bacterial pathogens.

## Materials and Methods

### Plasmids and Reagents

DNA for OspI was amplified from *Shigella flexneri* 2a strain 301 and cloned into the pGEX-6p-2 vector. The mutation C62A of OspI was generated by using the standard PCR method. Human Ubc13 was cloned to the pET14b vector with an N-terminal 6×His tag. The human E2 enzymes, UBE2T and UBE2L3, and their mutant were cloned into an adapted pGEX-4T-1 vector with a TEV cut site and a C-terminal 10×His tag. The OspI mutations for the pull-down assays were generated from the C62A construct. The OspI mutations for the deamidation assays were generated from the wild-type OspI construct. All Mutagenesis were performed with the QuickChange Site-Directed Mutagenesis Kit (Stratagene) and all plasmids were verified by DNA sequencing.

### Expression and Purification of Recombinant Proteins

All recombinant proteins were expressed in the *E. coli* BL21 (DE3) strain (Novagen) at 22°C for 12 h after 0.4 mM IPTG induction when OD600 reached 0.6–0.8. The C62A mutant of OspI was purified with glutathione-sepharose resin followed by the PreScission protease digestion. Further purification was performed with the Hitrap Q HP ion exchange and subsequent gel-filtration chromatography (GE Healthcare). Tag-free OspI proteins including wild type and its mutants used in the deamidation assays were purified as C62A. GST-fused C62A and its mutant proteins used in His-tag pull-down assays were purified with the glutathione-sepharose resin and gel filtration chromatography. GST tags of wild-type UBE2T and UBE2L3, and their mutant in which the α1 helix is replaced by the Ubc13 sequence were removed by the TEV protease digestion after GST affinity purification. Ubc13 proteins (wild type and mutants) were purified with Ni-NTA resin and gel filtration chromatography. All purification processes were performed at 4°C.

### Crystallization and Data Collection

To prepare OspI-Ubc13 complex, purified C62A was incubated with wild-type Ubc13 at the molar ratio of 1∶1 for 12 h at 4°C in the buffer containing 50 mM Tris-HCl pH 8.0 and 150 mM NaCl. The final concentration of the mixture was 20 mg/ml. In initial crystal screening, crystals of OspI-Ubc13 complex appeared after 7 days. After optimization, diffraction-qualified crystals were obtained after 12 h in the well condition containing 0.2 M Sodium thiocyanate, 20% PEG3350 and 0.1 M HEPES pH 7.0. Crystals of OspI-Ubc13 complex were transferred into the well solution supplemented with 15% glycerol as the cryoprotectant, and then flash-cooled into the liquid nitrogen. All crystallization experiments were carried out with the hanging-drop vapor diffusion method at 16°C. The diffraction data of OspI-Ubc13 complex was collected on the BL17U1 beamline at Shanghai Synchrotron Radiation Facility (SSRF). The diffraction data was processed by using the HKL-2000 package.

### Structural Determination and Refinement

The OspI-Ubc13 complex structure was solved with the molecular replacement method by using the wild-type OspI alone structure (pdb ID: 3B21) and the Ubc13 model from TRAF-UBC13 complex (pdb ID: 3HCT) as the searching modes and the Phaser program in CCP4 [44]. The final structure was refined to 2.3 Å with Rwork/Rfree of 21.04%/24.79% in CNS1.3 [45]. There is one 1∶1 OspI-Ubc13 complex in an asymmetric unit. Due to the lack of electron density, the N-terminal 20 residues of OspI, the N-terminal 6×His tag and the first methionine residue of Ubc13 are missed in the final model. The model building was performed in Coot [46]. The final structure was checked by the program Procheck [47]. All structural pictures were drawn in PyMol (http://www.delanoscientific.com/). Statistics of data collection and refinement are listed in Table 1.

### Pull-down Assay and Deamidation Assay

10 µg His-Ubc13 (wild type or mutants) was preloaded onto 5 µl Ni-NTA resin and then incubated with GST-fused C62A or its mutants at 4°C for 2.5 hours in the binding buffer containing 20 mM Tris-HCl pH 7.4, 150 mM NaCl, 20 mM imidazole, 0.5% NP-40 and 10% Glycerol. After extensive washing, the pull-down samples were loaded into SDS-PAGE and stained with Coomassie Brilliant Blue. The pull-down assays for wild-type UBE2T, UBE2T with the surface residues (M_1_QRAS_5_, R_9_ and M_13_) in the α1 helix replaced with those of Ubc13 (M_1_AGLPR_6_, K_10_ and R_14_), wild-type UBE2L3, and UBE2L3 with the exposed residues (M_1_AAS_4_ and E_13_) in the α1 helix replaced with those of Ubc13 (M_1_AGLP_5_ and R_14_) were performed similarly. For the deamidation assays, 10 µg Ubc13 proteins (wild type or mutants) were incubated with 7.16 ng (0.03 µM) wild-type OspI or its mutants at 30°C for the indicated time in the reaction buffer containing 20 mM Tris-HCl pH 7.4, 100 mM NaCl and 0.5 mM DTT. The samples were separated in native PAGE, stained with Coomassie Brilliant Blue and then quantified with the program ImageJ.

### Luciferase Reporter and Cell-free Assays

Luciferase activities were determined in HEK293 cells at 12 h after co-transfection with the NF-κB reporter plasmid (10 ng), Renilla construct (5 ng), TRAF6 (100 ng) and OspI variants (50 ng) by using the dual luciferase assay kit (Promega) according to the manufacturer's instructions. For cell-free assay for NF-κB signaling, U937 cell lysate were centrifuged at 100,000 g for 1 h to get the supernatant for preparing the S100 cell extract. 70 ng TRAF6 were incubated with 12 ng OspI mutants in the U937 S100 cell extract in the reaction buffer (50 mM Tris-Cl pH 7.5, 5 mM MgCl_2_, 2 mM ATP, 0.03 µM the phosphatase inhibitor Microcystin) at 30°C for 1 h. Samples were analyzed by immunoblotting and using anti-IκBα antibody.

### Ubiquitination Assay

Ubiquitination assays were carried out in 10 µl reaction buffer (20 mM Tris-HCl, pH 7.4, 2 mM ATP, 5 mM MgCl_2_ and 0.1 mM DTT). 200 ng E1, 500 ng Uev2, 500 ng Ubc13 and 70 ng TRAF6 were incubated with the indicated amount of C62A at 30°C for 1 h. Reactions were terminated with the SDS-PAGE sample buffer and analyzed by immunoblotting with anti-ubiquitin antibody.

### Infection Assay

In-frame deletion of *OspI* from *Shigella flexneri* 2a 2457T was performed as previously described [17]. The *OspI* mutant genes were cloned into the rescue plasmid pME6032 for expression in Δ*OspI Shigella*. For infection assay, HeLa cells were infected with the indicated *Shigella* strains at MOI of 100∶1, and centrifuged at 800 g for 10 min at room temperature to facilitate bacteria attachment. To detect IκBα phosphorylation, the cells were incubated with the bacteria at 37°C for 10 minutes. The collected cells were then lysed in 2× Laemmli sample buffer. The protein samples were subjected onto SDS-PAGE gel and immunoblotting analysis. To detect IL8 mRNA expression, the cells were incubated with the bacteria at 37°C for one hour.

### Quantitative RT–PCR Analysis

Total RNA was extracted by RNA extraction kit (Qiagen) and cDNA was generated with M-MLV reverse transcriptase (Promega). Real-time PCR was performed on Applied Biosystems 7500 Fast Real-Time PCR System using the SYBR Green system (TaKaRa). The primers used for quantitative RT–PCR analysis of human IL8 and human GAPDH have been verified in previous paper [18]. To calculate the relative expression fold, the human IL8 mRNA level was normalized to that of GAPDH.

### Secretion Assay

To test the secretion level of OspI mutant proteins, the C-terminal Flag-tagged *OspI* mutant genes were cloned into the rescue plasmid PME6032. The secretion assays were carried out by strictly following the previously described procedure [48]. The Δ*OspI Shigella* strains complemented with the Flag-tagged *OspI* mutant genes were cultured in BHI broth at 37°C. Aliquots of 2.5 ml of the *Shigella* cultures were washed with ice-cold PBS and resuspended in 1 ml PBS. After incubation at 37°C for 5 min, 3 µl 1% Congo red were added to the bacterial suspension, which was incubated for 10 min at 37°C and centrifuged at 14 000 g for 5 min at 4°C. The supernatant was passed through a 0.45 µm pore size filter, and trichloroacetic acid was added to the resultant supernatant (0.5 ml) at a final concentration of 6%. The secreted OspI mutant proteins present in PBS containing 0.003% Congo red were pelleted down at 14,000 g for 5 min at 4°C. Each pellet from the same number of bacteria was separated by 12% SDS–PAGE and immunoblotted with the anti-Flag antibody.

## Supporting Information

Figure S1 **OspI and Ubc13 Form a Heterodimeric Complex in Crystals.** The obtained crystals of the OspI-Ubc13 mixture were washed for more than 3 times by using the well solution and denatured in SDS sample loading buffer at 95°C for 10 minutes. The sample was loaded into SDS-PAGE and stained by Cossmassie Brilliant Blue.
(TIF)

Figure S2 **The Conformational Changes of Ubc13 upon OspI Binding.** (A) Two views of the conformational changes of Ubc13 upon OspI binding. The Ubc13 structures from Ubc13 alone (pdb ID: 1JBB), Ubc13-MMS2 (pdb ID: 1J7D), TRAF6-Ubc13 (pdb ID: 3HCT) and CHIP-Ubc13 complexes (pdb ID: 2C2V) are superimposed with the Ubc13 structure in the complex with OspI. The structures are colored and labeled as indicated. The conformational changes of the α1 helix were marked as the black arrows. (B) The Gln142 wedging into the cleft between Leu4 and Arg6 in the α1 helix of Ubc13 induces the redirection of Pro5, which is highlighted with a black arrow.
(TIF)

Figure S3 **Comparison of OspI with Structural Homologues.** (A) Crystal structure of Ubc13-bound OspI. (B–I) Crystal structures of OspI homologues, AvrPphB (B), CHBP (C), Cif (D), CYLD (E), USP7 (F), USP14 (G), USP8 (H) and USP21 (I). All structures represented as cartoon are labeled and colored as indicated. The core secondary structural elements of papain-fold in each structure are highlighted in dark purple. The OspI-like structural regions in the deubiquitinating enzymes, CYLD, USP7, USP14, USP8 and USP21, are indicated in black circles (E–I). (The pdb IDs of AvrPphB, CHBP, Cif, CYLD, USP7, USP14, USP8 and USP21 cited in this figure are 1UKF, 3EIR, 3EFY, 2VHF, 1NBF, 2AYO, 2GFO and 3I3T, respectively.)
(TIF)

Figure S4 **Similar Expression and Secretion Levels of OspI Mutant Proteins.** The C-terminal Flag-tagged OspI mutant proteins secreted from the indicated *OspI*-complemented *Shigella* strains by addition of Congo red (0.003% final concentration) were analyzed by immunoblotting with the anti-Flag antibody.
(TIF)

Figure S5 **Conformational Changes of OspI upon Ubc13 Binding and Structural Comparison of the Catalytic Residues of OspI and AvrPphB.** (A) Structural superimposition between Ubc13-bound (green) and wild-type free OspI (blue, pdb ID: 3B21). The residues and the secondary structures undergoing conformational changes are labeled as indicated. (B) Structural superimposition of the catalytic residues of wild-type OspI with that of AvrPphB (pdb ID: 1UKF). The catalytic residues of OspI and AvrPphB are shown as sticks, colored in blue and grey, respectively. Besides the catalytic triads, Gln162 of OspI and Asn93 of AvrPphB participating in formation of the oxyanion holes for catalysis [31] are also represented. The active site Cys62 in the wild-type alone OspI structure has three conformations, and cannot be superimposed with the active residue C98 of AvrPphB. (C) Structural superimposition of the catalytic residues of Ubc13-bound OspI with that of AvrPphB (pdb ID: 1UKF). The catalytic residues of Ubc13-bound OspI and AvrPphB are shown as sticks, colored in green and grey, respectively. After the structural reassembly upon Ubc13 binding, the catalytic triad (Ala62-His145-Asp160) of the Ubc13-bound OspI can be well superimposed with that of AvrPphB.
(TIF)

Figure S6 **The residues, Gly3, Pro5, Arg14, Arg102 and T103 of Ubc13 are Required for the Recognition and Deamidation by OspI.** (A) Effects of the indicated Ubc13 mutations on OspI interactions. The interactions of GST-C62A with the indicated Ubc13 variants were examined in His-tag pull-down assays. (B) Quantitative analysis of the deamidation activities of wild-type OspI on the mutant Ubc13 proteins. The percent product (Ubc13-E100) of the mutant Ubc13 proteins formed as a function of time by wild-type OspI is plotted. All assays were repeated more than 2 times.
(TIF)

Figure S7 **OspI, TRAF6, CHIP and OTUB1 Bind to the Same Surface Regions of Ubc13.** (A–D) Complex structures of OspI-Ubc13 (A), TRAF6-Ubc13 (B, pdb ID: 3HCT), CHIP-Ubc13 (C, pdb ID: 2C2V) and OTUB1-Ubc13 (D, pdb ID: 4DHI) are represented in the same orientation of Ubc13. Ubc13 in all complex structures are colored in purple. OspI, TRAF6, CHIP and OTUB1 are colored in green, blue, red and orange, respectively, and labeled as indicated.
(TIF)

Figure S8 **Inhibition of TRAF6-catalyzed Polyubiquitination and IκBα Phosphorylation by the OspI C62A Mutant.** (A) Inhibition of TRAF6-catalyzed polyubiquitination by the C62A mutant. Ubiquitination assays were carried out in 10 µl reaction buffer (20 mM Tris-HCl, pH 7.4, 2 mM ATP, 5 mM MgCl_2_ and 0.1 mM DTT). 200 ng E1, 500 ng Uev2, 500 ng Ubc13 and 70 ng TRAF6 were incubated with C62A at 30°C for 1 h. Reactions were terminated with SDS-PAGE sample buffer and analyzed by immunoblotting with anti-ubiquitin antibody. The recombinant C62A protein was titrated with four concentrations (0.003 µM, 0.03 µM, 0.31µM and 3 µM (716 ng)) in the ubiquitination assays. (B) Inhibition of TRAF6-induced phosphorylation of IκBα by C62A in U937 S100 cell extract. The cell-free assays were performed as Figure 3A. The concentrations of the recombinant C62A protein in the reactions were titrated with 2 µM, 0.7 µM, 0.2 µM and 0.08 µM (19 ng), respectively.
(TIF)

Figure S9 **Two Types of Secreted Glutamine Deamidases from Various Bacterial Pathogens.** (A–B) The CNF-C type of glutamine deamidases, CNF1 (A, pdb ID: 1HQ0) and BFL1 (B, pdb ID: 3TU8). The catalytic residues of CNF1 and BFL1 are shown as sticks in purple and labeled as indicated. (C–F) The papain-like type of glutamine deamidases, wild-type OspI (pdb ID: 3B21), CHBP (pdb ID: 3EIR), Cif (pdb ID: 3EFY) and PMT (pdb ID: 2EBF). Surface structure representations of these glutamine deamidases are placed at the right panels. The catalytic cysteine residues of the deamidases are highlighted in purple in the left cartoon structures and their positions in the surface structures (right) are marked with black arrows. The catalytic residue Cys62 of OspI harboring three conformations is buried in the glutamine-binding pocket (C) and covalently bound by a disulfide bond formed with Cys65 [18]. The catalytic cysteine residues, Cys156 of CHBP and Cys109 of Cif, are partially exposed at the edges of the glutamine-binding pockets [29], [40] (D–E). The catalytic residue Cys1165 of PMT is shielded in the deep glutamine-binding pocket and covalently bound by a disulfide bond formed with Cys1159 [41] (F). The glutamine-binding pockets of OspI, CHBP, Cif and PMT are indicated in black circles in the surface structures at the right panels.
(TIF)
