# Supplementary figures and images for: Complex Structure of OspI and Ubc13: The Molecular Basis of Ubc13 Deamidation and Convergence of Bacterial and Host E2 Recognition
Source: PLoS Pathog. 2013 Apr 25;9(4):e1003322. doi: 10.1371/journal.ppat.1003322 (PMC3636029; doi:10.1371/journal.ppat.1003322)

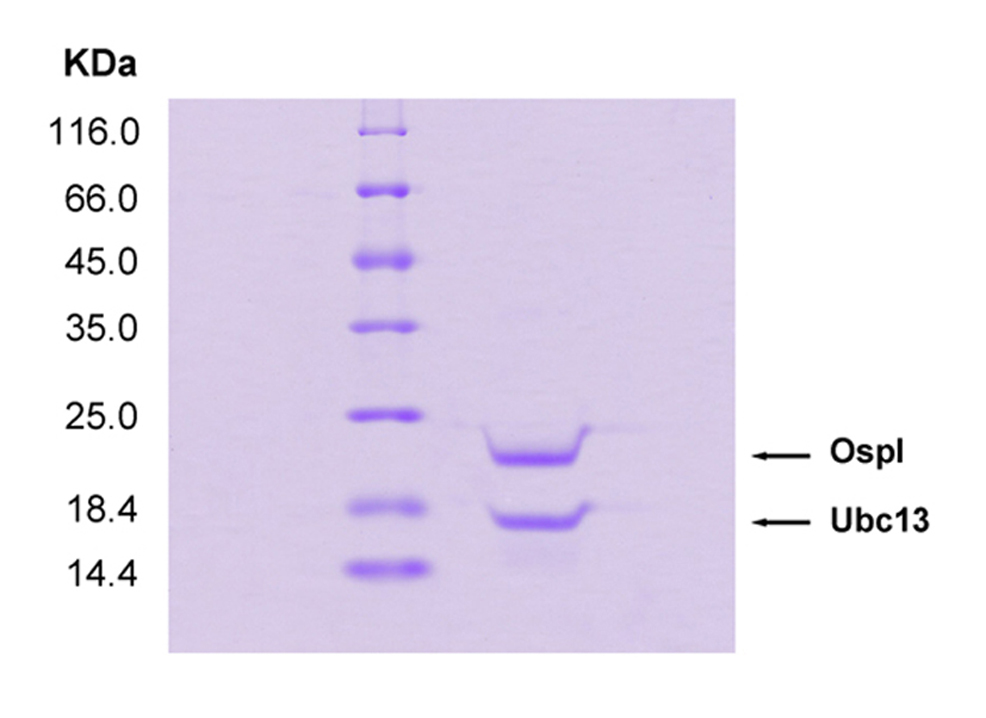

Supplement: Figure S1 — OspI and Ubc13 Form a Heterodimeric Complex in Crystals. The obtained crystals of the OspI-Ubc13 mixture were washed for more than 3 times by using the well solution and denatured in SDS sample loading buffer at 95°C for 10 minutes. The sample was loaded into SDS-PAGE and stained by Cossmassie Brilliant Blue. (TIF) [file ppat.1003322.s001.tif]

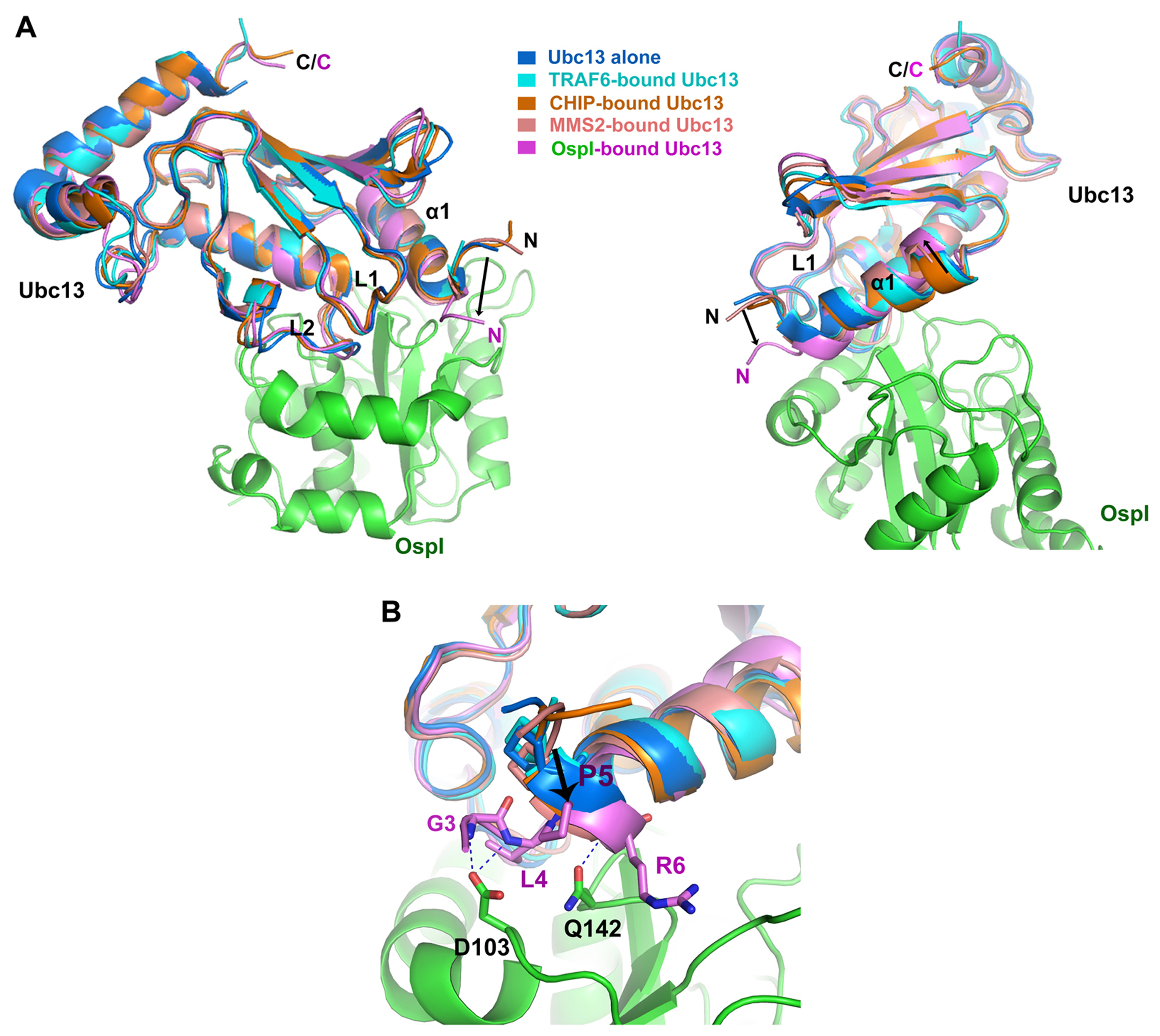

Supplement: Figure S2 — The Conformational Changes of Ubc13 upon OspI Binding. (A) Two views of the conformational changes of Ubc13 upon OspI binding. The Ubc13 structures from Ubc13 alone (pdb ID: 1JBB), Ubc13-MMS2 (pdb ID: 1J7D), TRAF6-Ubc13 (pdb ID: 3HCT) and CHIP-Ubc13 complexes (pdb ID: 2C2V) are superimposed with the Ubc13 structure in the complex with OspI. The structures are colored and labeled as indicated. The conformational changes of the α1 helix were marked as the black arrows. (B) The Gln142 wedging into the cleft between Leu4 and Arg6 in the α1 helix of Ubc13 induces the redirection of Pro5, which is highlighted with a black arrow. (TIF) [file ppat.1003322.s002.tif]

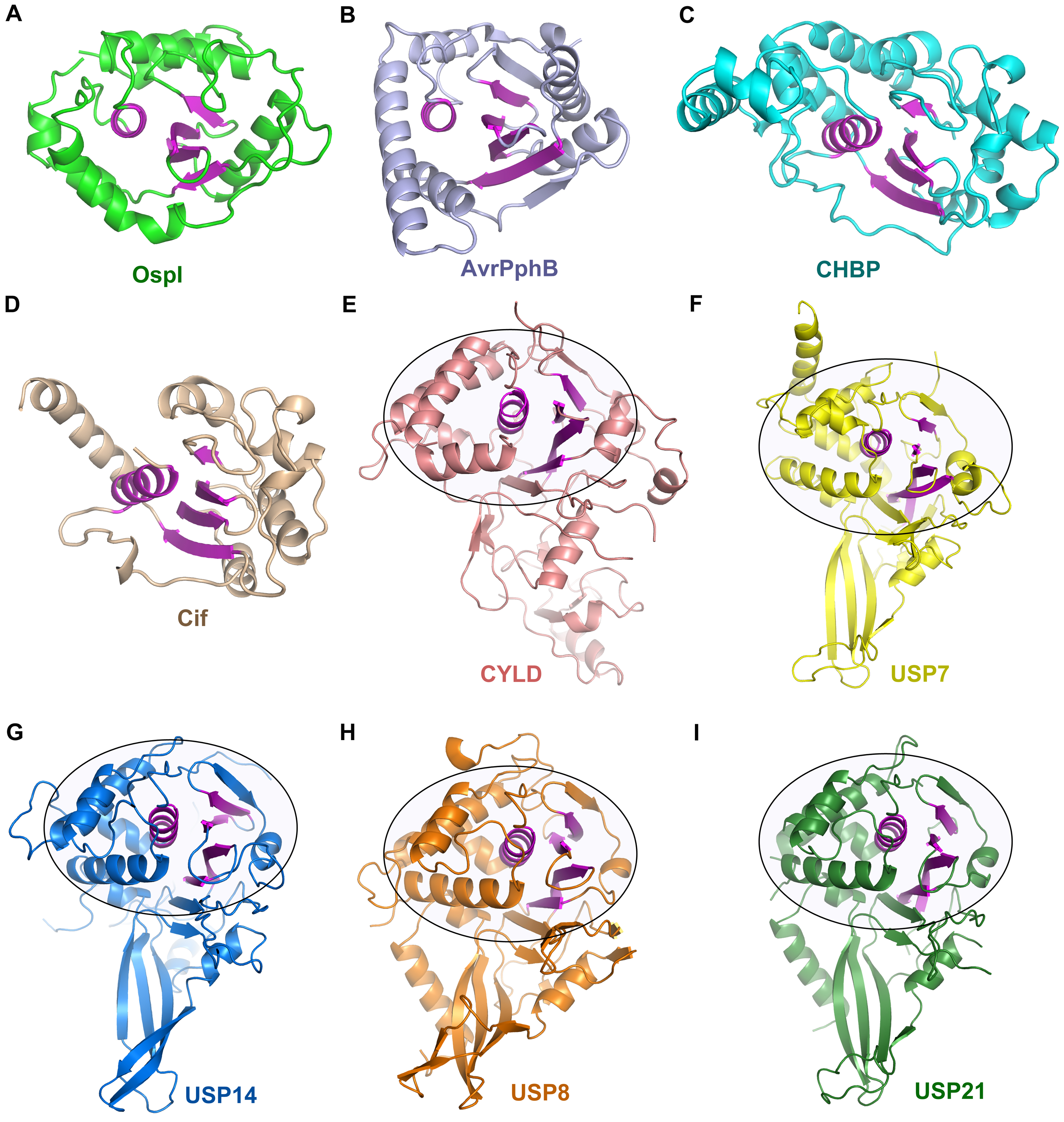

Supplement: Figure S3 — Comparison of OspI with Structural Homologues. (A) Crystal structure of Ubc13-bound OspI. (B–I) Crystal structures of OspI homologues, AvrPphB (B), CHBP (C), Cif (D), CYLD (E), USP7 (F), USP14 (G), USP8 (H) and USP21 (I). All structures represented as cartoon are labeled and colored as indicated. The core secondary structural elements of papain-fold in each structure are highlighted in dark purple. The OspI-like structural regions in the deubiquitinating enzymes, CYLD, USP7, USP14, USP8 and USP21, are indicated in black circles (E–I). (The pdb IDs of AvrPphB, CHBP, Cif, CYLD, USP7, USP14, USP8 and USP21 cited in this figure are 1UKF, 3EIR, 3EFY, 2VHF, 1NBF, 2AYO, 2GFO and 3I3T, respectively.) (TIF) [file ppat.1003322.s003.tif]

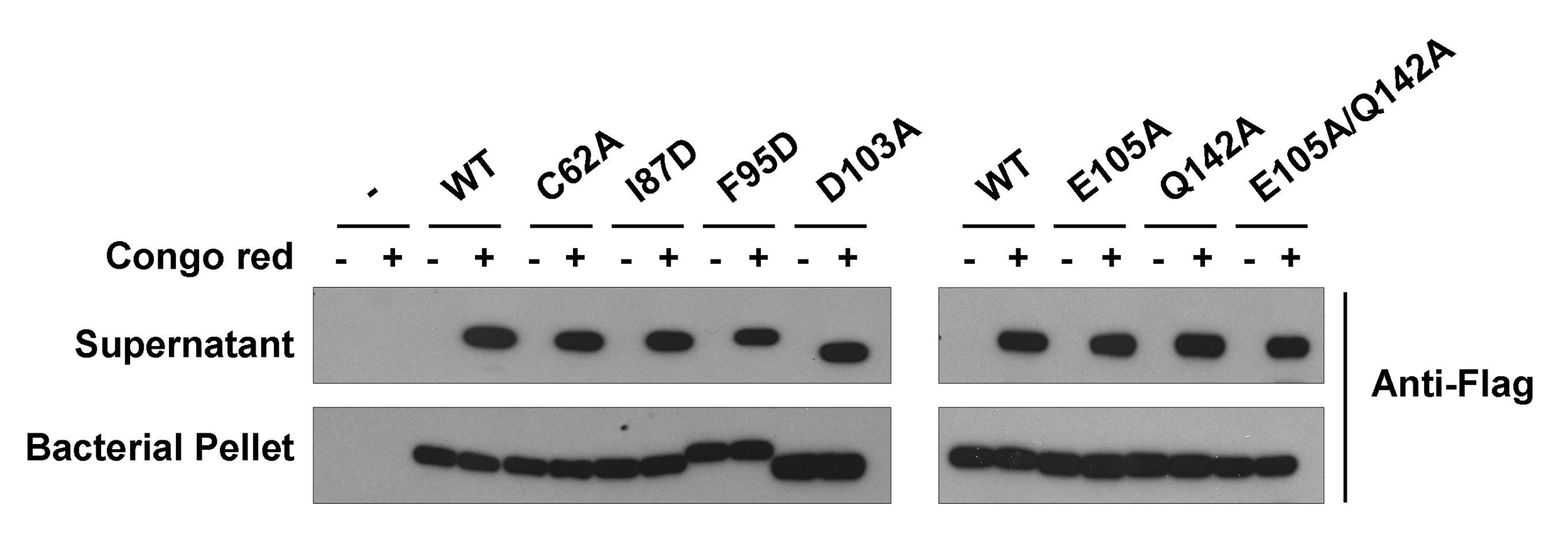

Supplement: Figure S4 — Similar Expression and Secretion Levels of OspI Mutant Proteins. The C-terminal Flag-tagged OspI mutant proteins secreted from the indicated OspI-complemented Shigella strains by addition of Congo red (0.003% final concentration) were analyzed by immunoblotting with the anti-Flag antibody. (TIF) [file ppat.1003322.s004.tif]

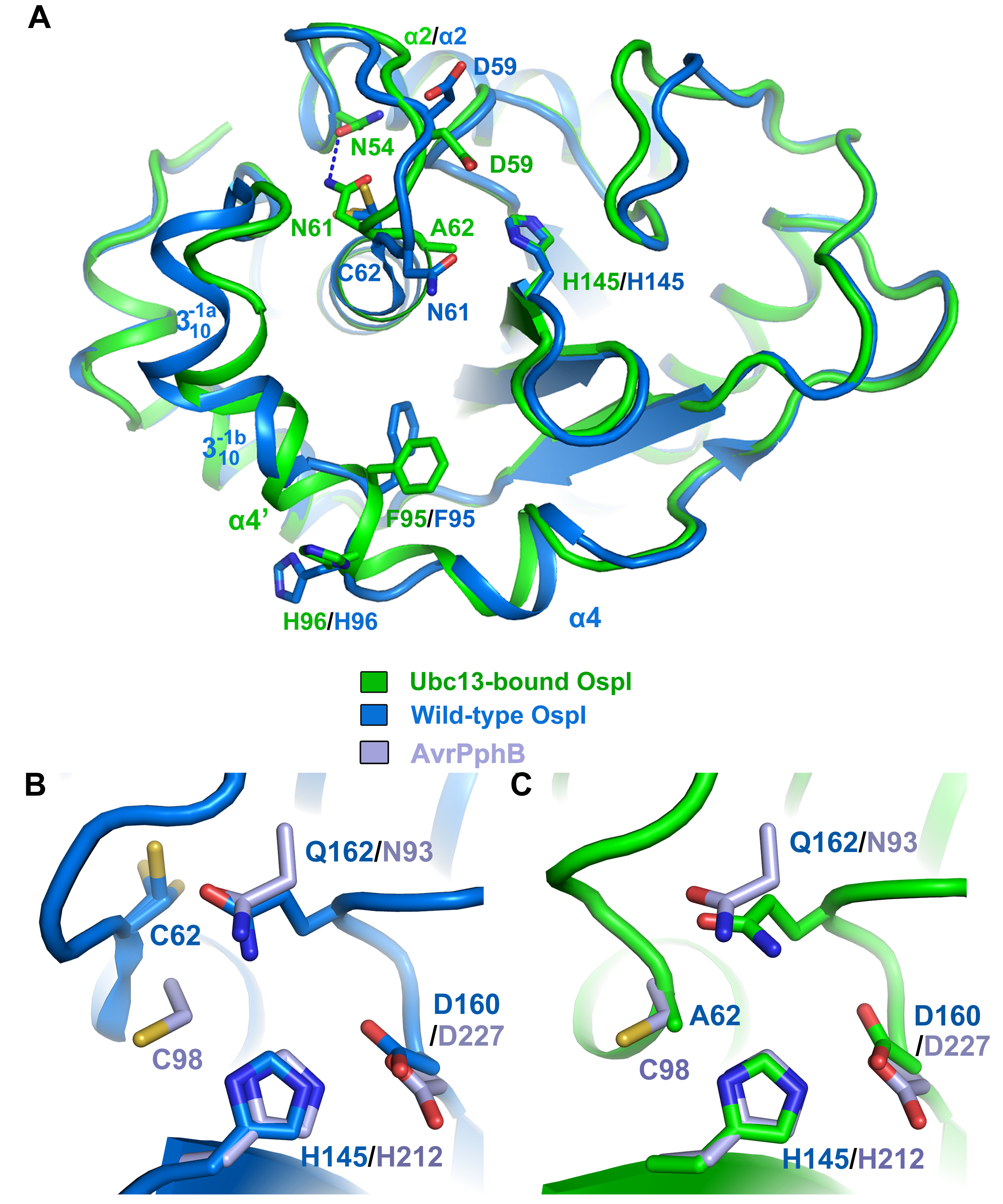

Supplement: Figure S5 — Conformational Changes of OspI upon Ubc13 Binding and Structural Comparison of the Catalytic Residues of OspI and AvrPphB. (A) Structural superimposition between Ubc13-bound (green) and wild-type free OspI (blue, pdb ID: 3B21). The residues and the secondary structures undergoing conformational changes are labeled as indicated. (B) Structural superimposition of the catalytic residues of wild-type OspI with that of AvrPphB (pdb ID: 1UKF). The catalytic residues of OspI and AvrPphB are shown as sticks, colored in blue and grey, respectively. Besides the catalytic triads, Gln162 of OspI and Asn93 of AvrPphB participating in formation of the oxyanion holes for catalysis [31] are also represented. The active site Cys62 in the wild-type alone OspI structure has three conformations, and cannot be superimposed with the active residue C98 of AvrPphB. (C) Structural superimposition of the catalytic residues of Ubc13-bound OspI with that of AvrPphB (pdb ID: 1UKF). The catalytic residues of Ubc13-bound OspI and AvrPphB are shown as sticks, colored in green and grey, respectively. After the structural reassembly upon Ubc13 binding, the catalytic triad (Ala62-His145-Asp160) of the Ubc13-bound OspI can be well superimposed with that of AvrPphB. (TIF) [file ppat.1003322.s005.tif]

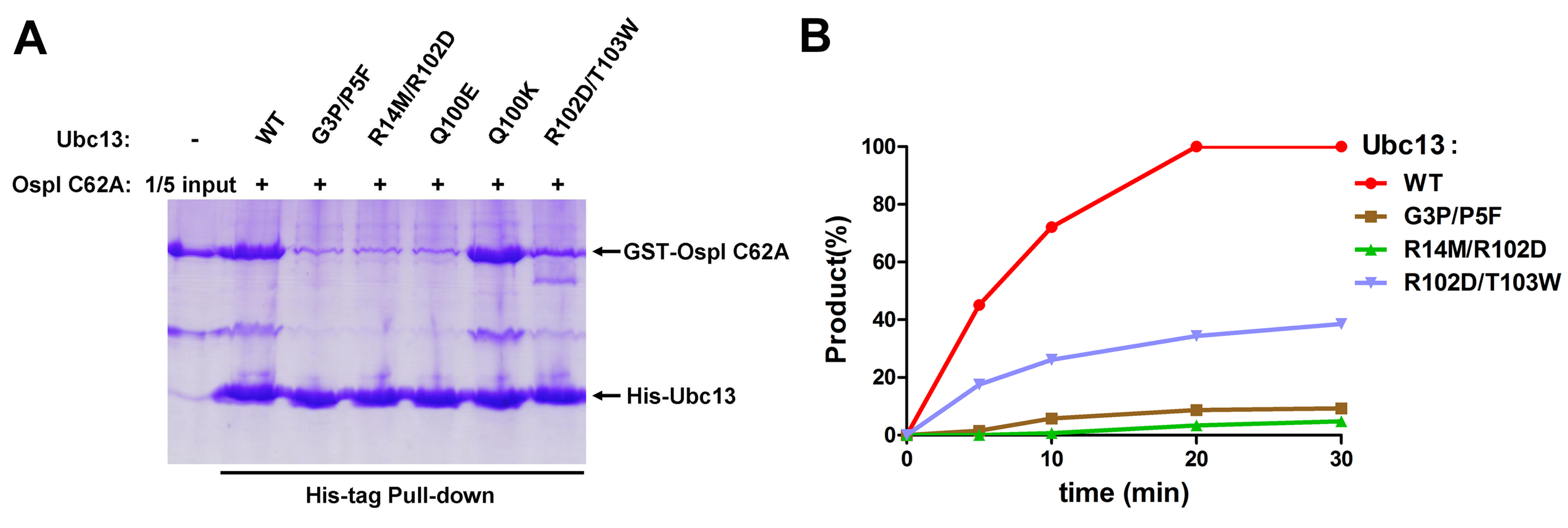

Supplement: Figure S6 — The residues, Gly3, Pro5, Arg14, Arg102 and T103 of Ubc13 are Required for the Recognition and Deamidation by OspI. (A) Effects of the indicated Ubc13 mutations on OspI interactions. The interactions of GST-C62A with the indicated Ubc13 variants were examined in His-tag pull-down assays. (B) Quantitative analysis of the deamidation activities of wild-type OspI on the mutant Ubc13 proteins. The percent product (Ubc13-E100) of the mutant Ubc13 proteins formed as a function of time by wild-type OspI is plotted. All assays were repeated more than 2 times. (TIF) [file ppat.1003322.s006.tif]

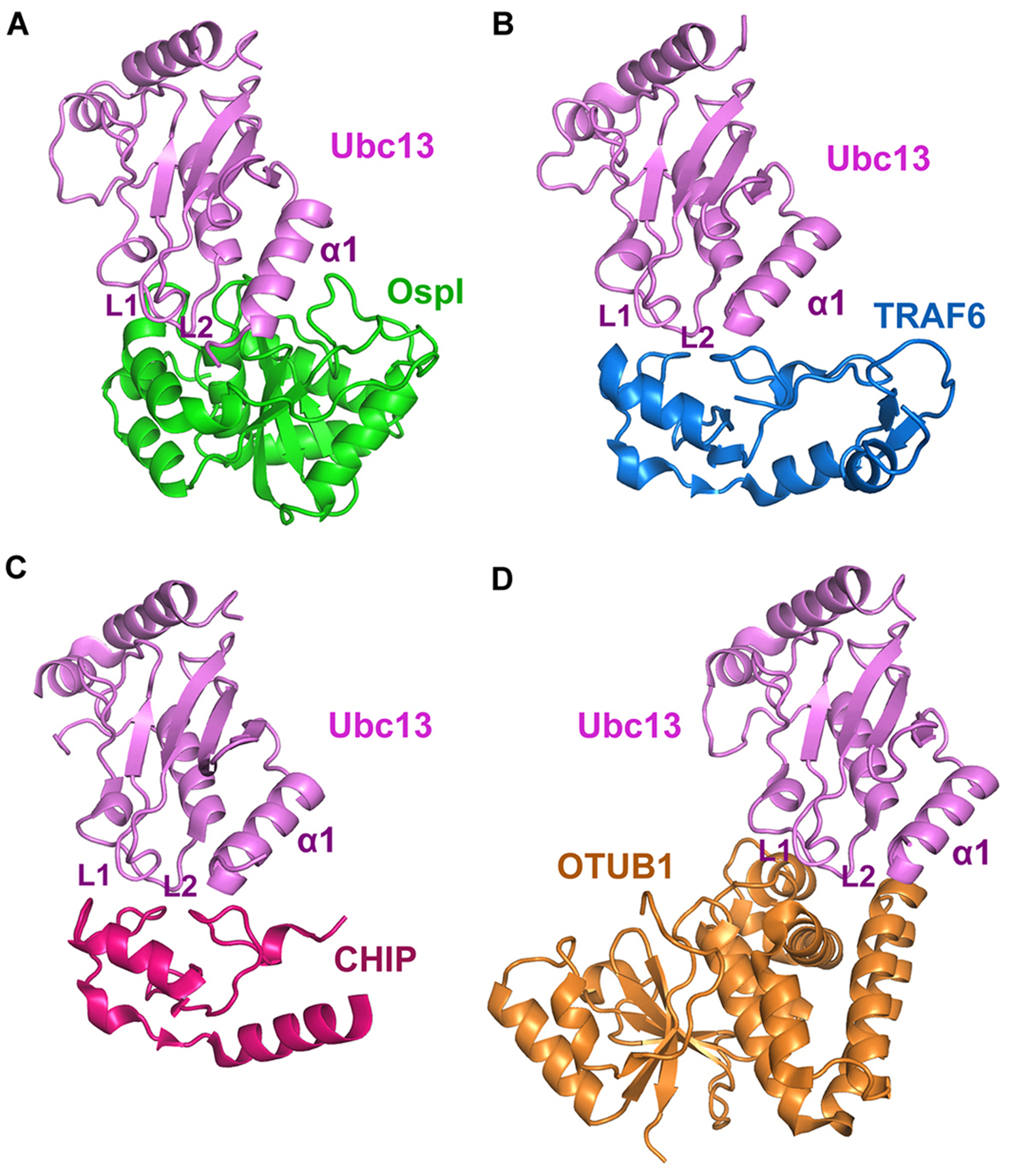

Supplement: Figure S7 — OspI, TRAF6, CHIP and OTUB1 Bind to the Same Surface Regions of Ubc13. (A–D) Complex structures of OspI-Ubc13 (A), TRAF6-Ubc13 (B, pdb ID: 3HCT), CHIP-Ubc13 (C, pdb ID: 2C2V) and OTUB1-Ubc13 (D, pdb ID: 4DHI) are represented in the same orientation of Ubc13. Ubc13 in all complex structures are colored in purple. OspI, TRAF6, CHIP and OTUB1 are colored in green, blue, red and orange, respectively, and labeled as indicated. (TIF) [file ppat.1003322.s007.tif]

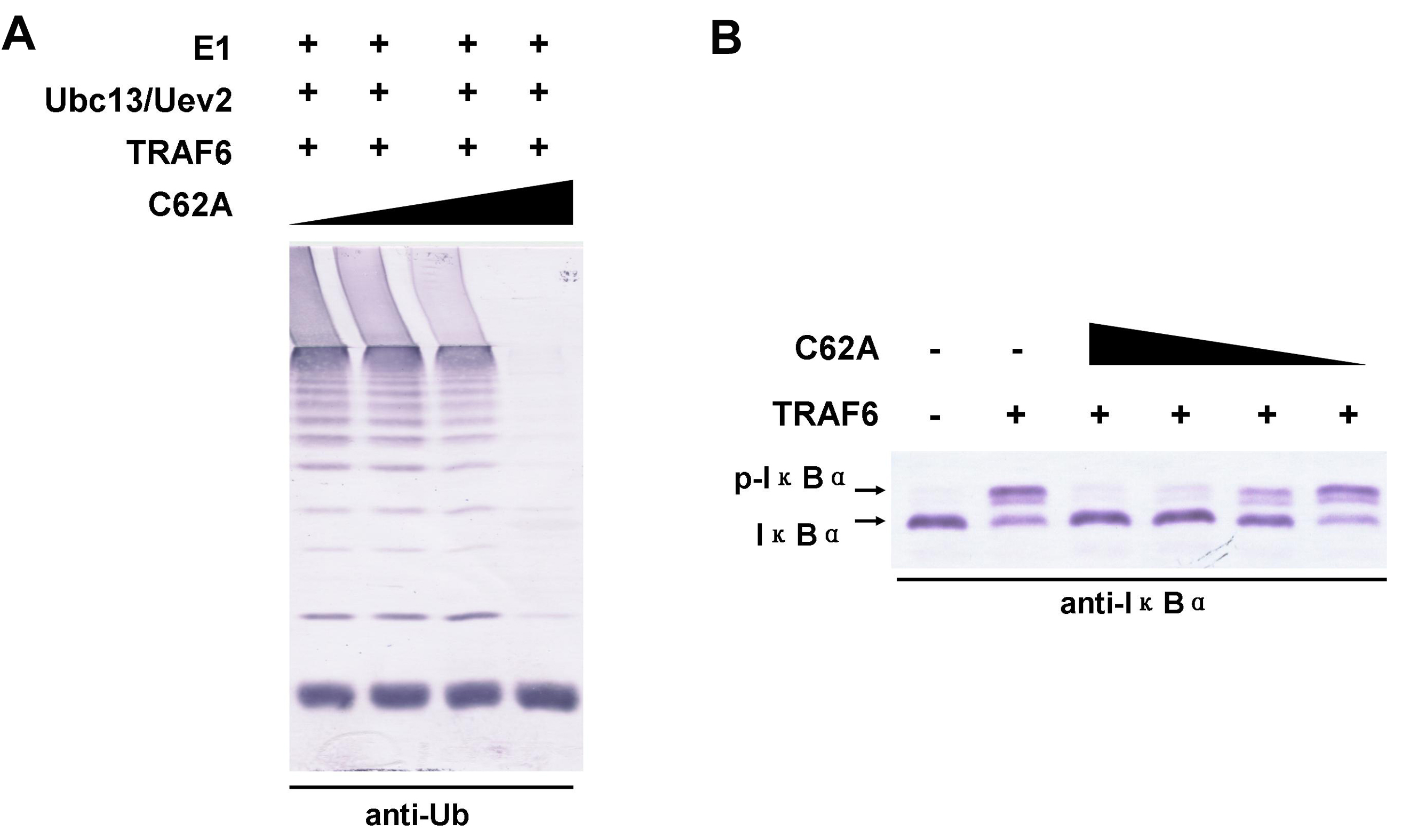

Supplement: Figure S8 — Inhibition of TRAF6-catalyzed Polyubiquitination and IκBα Phosphorylation by the OspI C62A Mutant. (A) Inhibition of TRAF6-catalyzed polyubiquitination by the C62A mutant. Ubiquitination assays were carried out in 10 µl reaction buffer (20 mM Tris-HCl, pH 7.4, 2 mM ATP, 5 mM MgCl2 and 0.1 mM DTT). 200 ng E1, 500 ng Uev2, 500 ng Ubc13 and 70 ng TRAF6 were incubated with C62A at 30°C for 1 h. Reactions were terminated with SDS-PAGE sample buffer and analyzed by immunoblotting with anti-ubiquitin antibody. The recombinant C62A protein was titrated with four concentrations (0.003 µM, 0.03 µM, 0.31µM and 3 µM (716 ng)) in the ubiquitination assays. (B) Inhibition of TRAF6-induced phosphorylation of IκBα by C62A in U937 S100 cell extract. The cell-free assays were performed as Figure 3A. The concentrations of the recombinant C62A protein in the reactions were titrated with 2 µM, 0.7 µM, 0.2 µM and 0.08 µM (19 ng), respectively. (TIF) [file ppat.1003322.s008.tif]

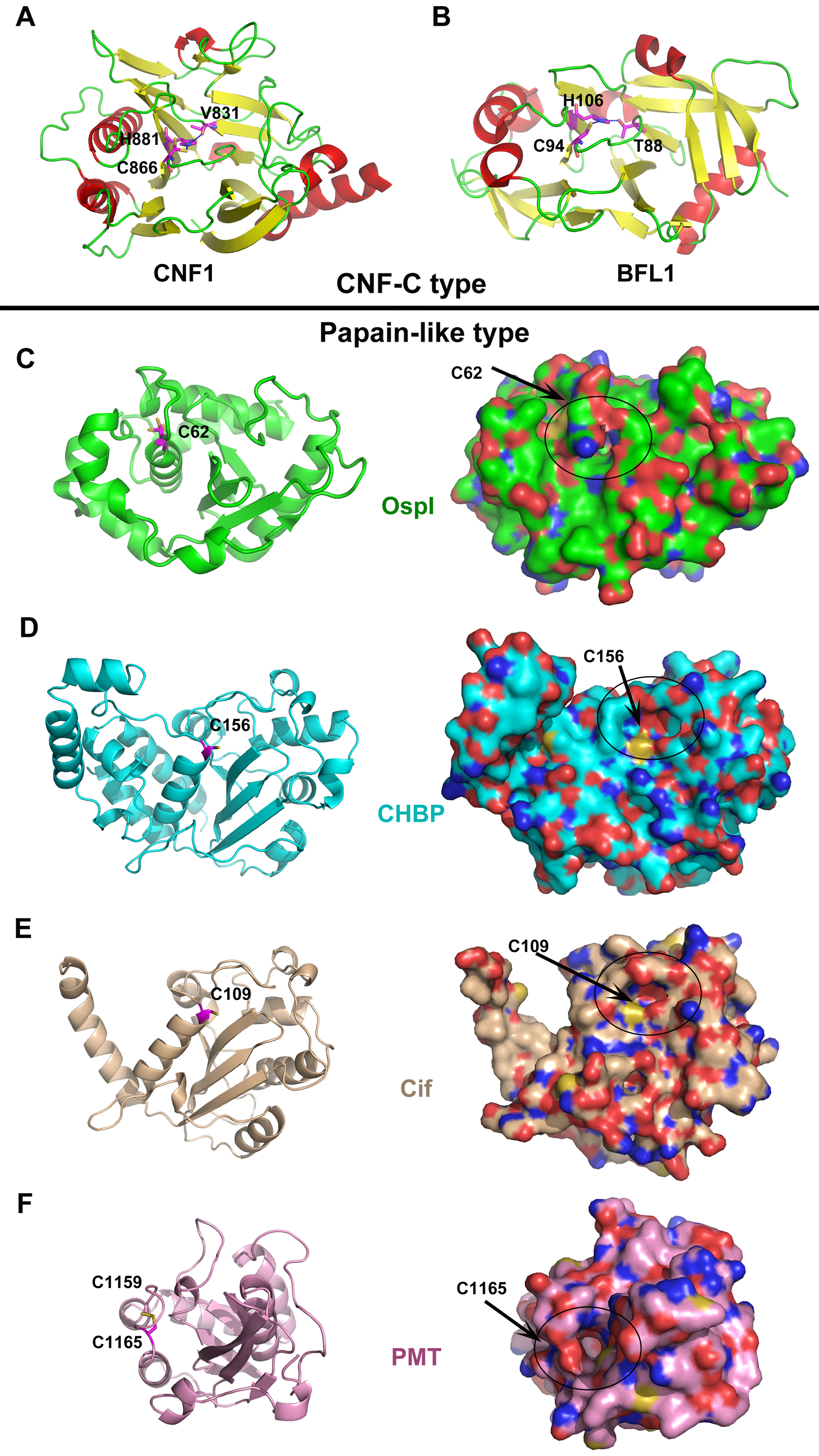

Supplement: Figure S9 — Two Types of Secreted Glutamine Deamidases from Various Bacterial Pathogens. (A–B) The CNF-C type of glutamine deamidases, CNF1 (A, pdb ID: 1HQ0) and BFL1 (B, pdb ID: 3TU8). The catalytic residues of CNF1 and BFL1 are shown as sticks in purple and labeled as indicated. (C–F) The papain-like type of glutamine deamidases, wild-type OspI (pdb ID: 3B21), CHBP (pdb ID: 3EIR), Cif (pdb ID: 3EFY) and PMT (pdb ID: 2EBF). Surface structure representations of these glutamine deamidases are placed at the right panels. The catalytic cysteine residues of the deamidases are highlighted in purple in the left cartoon structures and their positions in the surface structures (right) are marked with black arrows. The catalytic residue Cys62 of OspI harboring three conformations is buried in the glutamine-binding pocket (C) and covalently bound by a disulfide bond formed with Cys65 [18]. The catalytic cysteine residues, Cys156 of CHBP and Cys109 of Cif, are partially exposed at the edges of the glutamine-binding pockets [29], [40] (D–E). The catalytic residue Cys1165 of PMT is shielded in the deep glutamine-binding pocket and covalently bound by a disulfide bond formed with Cys1159 [41] (F). The glutamine-binding pockets of OspI, CHBP, Cif and PMT are indicated in black circles in the surface structures at the right panels. (TIF) [file ppat.1003322.s009.tif]
